# A measure of the concentration of rare events

**DOI:** 10.1038/srep32369

**Published:** 2016-08-31

**Authors:** Rafael Prieto Curiel, Steven Bishop

**Affiliations:** 1 University College London, Mathematics Department, London, WC1E 6BT, United Kingdom

## Abstract

We introduce here an index, which we call the Rare Event Concentration Coefficient (*RECC*), that is a measure of the dispersion/concentration of events which have a low frequency but tend to have a high level of concentration, such as the number of crimes suffered by a person. The Rare Event Concentration Coefficient is a metric based on a statistical mixture model, with a value closer to zero meaning that events are homogeneously distributed, and a value closer to one meaning that the events have a higher degree of concentration. This measure may be used to compare the concentration of events over different time periods and over different regions. Other traditional approaches for the dispersion/concentration of a variable tend to be blind to structural changes in the pattern of occurrence of rare events. The *RECC* overcomes this issue and we show here two simple applications, first by using the number of burglaries suffered in Netherlands and then by using the number of volcanic eruptions in the world.

In many different practical contexts, being able to determine a measure of the degree of concentration of a variable is particularly useful. For instance, in the case of wealth distribution, the Gini coefficient has been used in many studies in Economical and Political Sciences. The Gini coefficient is a single valued number which works as a summary for the whole distribution and it helps us to determine whether a country is moving into a more egalitarian distribution of income or if its disparity is increasing, thus in a way it is a summary statistic which helps us compare different regions and over different time periods.

Similar comparisons are desirable in alternative contexts, for example, is crime more or less concentrated in certain regions after the introduction of surveillance systems in a city? Are road accidents more spatially disperse in Paris compared to Frankfurt? Are the number of claims that an insurance company receives from their customers being more concentrated this year? The data for these type of question has two characteristics which make it hard to deal with: it is both rare and also highly concentrated. In all these examples, the majority of the observations are equal to zero, but then, if a particular observation is not zero, then it is likely that the actual number is not small; so for instance, many accidents happen at the same place, leading to an accident black spot.

This phenomenon has been studied in different settings; in the case of crime, for example, although a high number of people or houses, in fact, suffer no crimes, those who suffer crime have an increased risk of suffering subsequent crimes^1^ and as a result, the majority of the observations are equal to zero but then, some observations are far away from being zero^2^. Another example comes from the study of human mobility patterns, where it has been studied by tracing the consecutive sightings of nearly half-a-million bank notes and also, by following 100,000 mobile users, that most of the individuals travel only over short distances^3^, which means that an individual is likely to be found only in a handful of different places and, if a threshold was placed on travel, most journeys would fall below the threshold.

Having a measure of the degree of concentration or dispersion of such events is useful since sometimes interventions (such as a policing strategy in the case of crime, or a road intervention in the case of road accidents) might result in the displacement of such events, rather than a genuine reduction. However, traditional measures of the concentration of a variable (such as the Gini coefficient or the entropy), fail to work as a tool to compare different levels of the concentration or to track structural changes in the way that these events happen, due to their extreme small frequency and their high levels of concentration.

## Counting data and its distribution

Here we focus on a counting process, that is, we select a variable that reflects the number of events with a certain property. Let *X*_*i*_ be the number of events that occurred over a fixed time interval, counted over some set *i* = 1, 2, …, *N*, referred to as *individuals*. This could be, for example, the number of burglaries suffered by the *i*-th household during the period of one year, say, or the number of insurance claims from the *i*-th customer or postcode. Assuming that having one unit of these events does not affect future probabilities of having any additional units, the number *X*_*i*_ follows a Poisson distribution with rate *λ*_*i*_. Under this assumption, the number *X*_*i*_ becomes one observation from a Poisson distribution, which means that if *X*_*i*_ is small or even zero, it could be the result of a small rate, but it could also be (with small probability perhaps) the result of a large rate and it was just good luck, or vice-versa in the case that *X*_*i*_ is large. If a person suffered zero crimes last year it does not mean that their rate is zero and they will never suffer crime.

We assume also that *X*_*i*_ is independent to *X*_*j*_ for *i* ≠ *j*, which might be a strong assumption for the particular context under consideration and needs to be fully examined before moving to the following step. In the case of crime, for example, the assumption of independence is perhaps valid only for large populations. Now, if there is a way in which we can collect the *N* individuals into *k* ≥ 1 distinct groups, where group *j* say, has *Q*_*j*_ individuals (or equivalently, has a relative size *q*_*j*_ = *Q*_*j*_/*N*), which have the same rate *λ*_*j*_, with *j* = 1, 2, …, *k*. Each one of the *N* individuals of the whole population belongs to one and only one group, so that *Q*_1_ + *Q*_2_ +⋯ + *Q*_*k*_ = *N* (or written in terms of the relative size *q*_1_ + *q*_2_ + ⋯ + *q*_*k*_ = 1). To avoid ambiguous definitions, we order the groups by their rate in increasing order, so *λ*_1_ < *λ*_2_ < ⋯ < *λ*_*k*_. This type of model is known as a mixture model^4^. If we consider a random individual from the population, the distribution of *X*_*i*_ might be expressed as


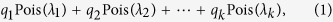


which means that the individual is allocated into the *j*-th group (with probability *q*_*j*_) and then has a Poisson distribution with the corresponding rate *λ*_*j*_.

The number of groups *k* is crucial for the mixture model. An easy (but useless) solution is to assign each individual to a different group, however, solutions with larger numbers of groups are less useful since for each additional group, its size and its rate need to be estimated, so this increases the number of parameters of the model. The (non-parametric) maximum likelihood estimator (*mle* or *npmle*) helps us compare between models with different number of groups, *k*, and to pick the best (in some sense) amongst them^5^ since in our case we have no prior information on the number of groups^6^. Other techniques to estimate the number of groups, using bootstrapping for example, are also available^7^. The model can be easily fitted using the statistical package CAMAN (Computer Assisted Analysis of Mixtures)^8^ in R^9^ by considering the observed *X*_*i*_, with *i* = 1, 2, …, *N*^10^.

The results obtained are: an estimate of the number of population groups 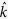, the corresponding rate for each group 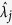, so that the collection of the rate of each group can be viewed as a vector 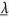, and the relative size of each population group 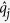, also expressed as a vector as 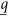. A goodness of fit test can help us accept or reject the distribution obtained^10^. A similar procedure using a mixture model has been used in different scenarios^4^, such as road accidents, mapping hepatitis B in Berlin^11^ and many more examples in epidemiology^5^.

Two special cases are interesting from the mixture model. First, if 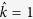 then this means that the best way to explain the observations is simply as a Poisson process with rate 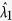, which is a homogeneous distribution over the whole population. The second case is when 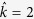 and 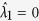, which means that the population can be divided into two groups, the first group has a rate equal to zero while the other group has a non-zero rate, which is a model known also as a Zero-Inflated Poisson Model^4^. Both scenarios, the homogeneous distribution and the Zero-Inflated Poisson Model, might be the result obtained from the mixture model.

The distribution of the rates 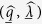 is powerful by itself since we can use these to simulate different observations under that distribution so that we can understand the natural departures from the distribution. However, the Rare Event Concentration Coefficient works as a summary statistic based on that distribution.

## Concentration metric

The Rare Event Concentration Coefficient (*RECC*) is defined in terms of the distribution of the rates 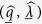 given by


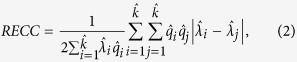


which is the Gini coefficient applied to the distribution of the rates. The Lorenz curve^12^ and the Gini coefficient^13^ of a distribution are often used as a measure of the concentration or dispersion of a variable, and so here we apply them to the mixture model. It is important to note that it is not the Gini coefficient computed directly from the observations *X*_*i*_, but rather the Gini coefficient of the distribution of the rates 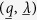. A value of the Gini coefficient closer to zero is interpreted as the process being more homogeneously distributed across the population, and a value closer to one means that the process is more concentrated in some population groups.

The Lorenz curve and the corresponding Gini coefficient of the distribution of the individual rates are comparable between different time periods and over different regions, even in the case in which the number of individuals changes from one region to the other, or the total number of events of the process changes. With this simple tool, we can compare the rates of processes in which there is randomness involved, and we can determine a useful metric for the concentration of events which are rare and tend to be highly concentrated.

## Relevant scenarios

Two relevant cases might be obtained from the *RECC*. The first scenario, if the *RECC* = 0 then this means that the process is homogeneously distributed across the entire population so that every individual has the same rate 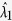. This scenario might happen even when the individuals have different observations *X*_*i*_ since here we consider the distribution of the rates and not the actual numbers *X*_*i*_.

The second scenario is the case when from data obtained is a Zero-Inflated Poisson Model (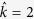 and 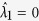). In such a case, the Rare Event Concentration Coefficient gives 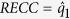, the relative size of the group which has a zero rate.

## Demonstration

Here, to demonstrate its use, we consider the number of burglaries suffered per household, obtained from a victimisation survey called the 1993 Police Monitor in Netherlands. The data has been used before as a test bed for analysis and to explore the level of concentration of burglary in that country and it contains a discussion on how data was obtained^14^. The number of houses that suffered 0, 1, 2 or more crimes is displayed in Table 1.

Burglaries are highly concentrated. From the population surveyed, 91.9% suffered no burglaries, but then from the houses that in fact suffered a burglary, 21% suffered more than one. The reasons why a particular house suffers more crime than others has been studied before (refs 1,15, 16, 17).

The mixture model applied to the number of burglaries suffered in the Netherlands gives as results 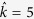, with sizes and rates displayed in Table 2.

The results of the mixture model applied to this data show that the population can be divided into five groups, the largest one (58.2% of the population) suffers no crime (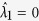), the second largest one (24.3%) suffers a rate of 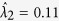, so that among any 9 houses of that group they expect to have experienced a burglary, and so on. There is a group (less than 3.3 out of 10,000 houses) which experiences a rate of almost 8 crimes per year (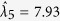).

**Figure 1:**
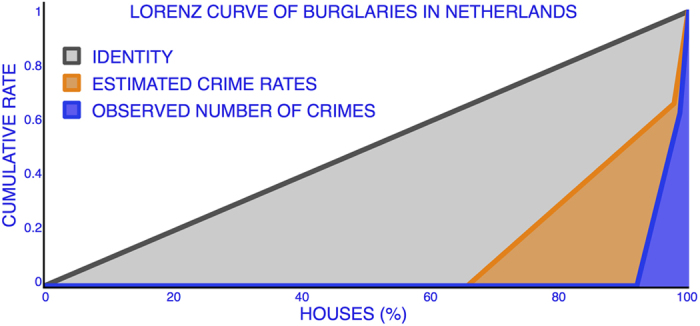
Lorenz curve of the individual crime rates and the number of crimes for Netherlands in 1993.

The Rare Event Concentration Coefficient gives *RECC* = 0.7643 and the Lorenz curve of the observed number of crimes and the estimated rate is displayed in Fig. 1 for comparison purposes. It is relevant to notice that the distribution of the rates 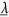 is much more evenly distributed than the actual crime and this is usually the case since, for example, the mixture model tells us that 24.3% of the population (Group 2) suffers a rate of 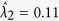, so that within the population from that group we expect only one victim from every 9 houses. In that group, the observed burglaries are highly uneven (for each house victimised there are eight houses not victimised), but the rates are uniformly distributed. The main element here is that the events considered are rare, so most of the observations (nearly 92% in the example from Netherlands) are zero, but it does not mean that their rate is zero.

If for some reason, the number of households which suffer 6 or more burglaries drops to zero (a change in only 17 out of the 39,849 observations) the *RECC* would be 0.7092, which means a difference of 0.0551. With the traditional approach to the concentration of a variable, if we compute the Gini coefficient directly to the number of crimes that each household suffered its value is 0.9362 and again, if for some reason the number of households which suffer 6 or more burglaries drops to zero, the Gini coefficient of the number of crimes suffered would be 0.9351, which only means a difference of 0.0011; an almost negligible change.

Naturally, if the number of households that suffer 6 or more crimes drops to zero, the change in terms of the number of crimes might not be significant, but it is relevant in terms of the structural change in the way that crime is suffered, and it is a change that would not be detected by the traditional Gini coefficient. However, our new metric would allow us to detect such change, even in the case when it occurred for such a small population group.

## Volcanic Eruptions

Another application of the *RECC* comes from the study of volcanic eruptions. Information about the location the 1,532 different volcanoes in the world and their eruptions is available^18^ and here we consider the number of confirmed eruptions for each volcano between 1966 and 2015 (50 years of confirmed eruptions), giving us a total of 1,746 eruptions.

Are volcanic eruptions a rare and concentrated event? In our context, out of the 1,532 different volcanoes, only 315 (around 21%) had an eruption in the last 50 years, yet, those volcanoes which had an eruption in the past 50 years, had on average 5.5 eruptions, meaning that volcanic eruptions are relatively rare and highly concentrated. The number of volcanoes which had 0, 1, 2 or more eruptions between 1966 and 2015 is displayed in Table 3.

Results of the mixture model applied to the volcanic eruptions gives a total of 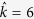 groups, so that the 1,532 volcanoes are grouped in an optimal way into 6 groups; the first one has an eruption rate of 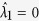 and a relative size 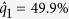, so that nearly half of the volcanoes are not expected to have an eruption. The second group has an eruption rate of 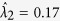 and a relative size 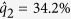, which means that nearly one-third of the volcanoes expect to have an eruption every 287 years. The group with the highest eruption rate has an eruption rate of 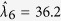 with a relative size of 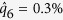, meaning that volcanoes within that group expect to have an eruption every 16.6 months. For volcanic eruptions, the *RECC* = 0.883.

**Figure 2:**
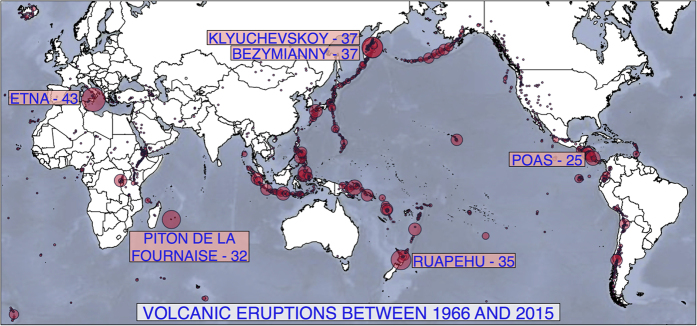
Distribution of the volcanic eruptions between 1966 and 2015. The size represents the number of eruptions. Figure made with Natural Earth, free vector and raster map data naturalearthdata.com (downloaded in August 2015), data from the Global Volcanism Program^19^ (downloaded in June 2016) and R (refs 9, 22 and 23).

The distribution of volcanoes throughout the world is highly similar to the positioning of the major tectonic belts and so many of the major volcanoes are clustered^19^, as shown in Fig. 2. For example, the three most active volcanoes during the past 50 years were Etna (with 43 eruptions), in Sicily, Italy; Bezymianny (with 37 eruptions) and Klyuchevskoy (also with 37 eruptions), both in Kamchatka, Russia. Additionally, volcanoes include a variety of cones and craters and some features are destroyed by continuing eruptions^18^, which raises the question of how we deal with observations that might be highly correlated? For example, Bezymianny and Klyuchevskoy are located at a distance of 9.7 kilometres and so in that small region, there was a total of 74 volcanic eruptions in the past 50 years.

Clustering volcanoes which are at a distance smaller than 10 kilometres into *volcanic regions* allows us to deal with the problem of correlated observations. By considering volcanic regions, so that Bezymianny and Klyuchevskoy in Kamchatka fall into a single region, instead of the 1,532 volcanoes, we obtain 1,439 regions with volcanoes, and by taking into account the number of eruptions from each region we can compute the mixture model and the corresponding *RECC*. By following this procedure, the number of regions changes, the largest *X*_*i*_ changes (from 43 eruptions of Mount Etna to 74 eruptions in the Kamchatka region) and the mixture model also changes. However, when we consider the 10 kilometre regions, the *RECC* changes from 0.883 to 0.879 and even to a value of 0.870 when clustering volcanoes into the considerably large regions with a radius of 20 kilometres.

By grouping observations which have a potential statistical dependence based on a physical attribute, such as nearby volcanoes or crimes separated in space within 200 metres^20^, or by taking into account burglaries that occur in the same block or neighbourhood, we obtain groups/regions for which the assumption of independence is fairly reasonable. Thus, the *RECC* is considerably stable when correlated observations are grouped based on a physical attribute.

## Human Mobility Patterns

Another area in which the *RECC* might be useful is in the study of Human Mobility Patterns. As it was studied, heterogeneity suggests that individuals might move following a Lévy flight^3^. Different research scenarios have been used, for example by following a large number of mobile users and by recording their position each they interact with his or her mobile or recording the position every given time period. The phone towers divide the region into a Voronoi lattice and the data set provides the closest tower to a user so that the location is only recorded by the nearest tower routeing which provides the communication service.

Now, the number of times that a particular mobile user is recorded inside a tower vicinity gives us an ideal setting for our study. It is reported^3^, for example, that from 186 measurements taken from a user, he or she was found inside only in 12 different tower vicinities, from which 96 (51.6%) and 67 (36.0%) occasions happened in the two most preferred locations. The pattern of that person shows that nearly 90% of his or her time is spent in two locations and their neighbouring regions, most likely his or her house and his or her office. In a similar study, some users were found who visit a much higher number of different vicinities^21^, and the frequency in which they move through different vicinities let us determine their mobility patterns.

By counting the number of times that a user is recorded in different tower vicinities, allows us to compare different mobility patterns that users might have. The *RECC* of the tower vicinities counts of different users gives us a way to compare their levels of mobility and for example, a smaller *RECC* implies that a user has a higher degree of mobility that a person who has a larger *RECC*. A larger *RECC* indicates that the person tends to move on a day-to-day basis only through a small number of neighbours of their home city. In terms of human mobility, the *RECC* takes in account the (potentially) highly concentrated nature of the regions in which a person moves, but also a random component which might motivate a person to visit places which he or she does not regularly visit.

## Discussion

The Rare Event Concentration Coefficient *RECC* based on the mixture model help us compare the concentration rate of events which are not frequent and tend to be highly concentrated by taking into account the random nature of such events. Other measurements which are traditionally used for the concentration/dispersion are meaningless since they do not detect structural changes of the process or they cannot be used to compare different regions or time intervals.

Using the example of the number of burglaries in Netherlands, we see that the Gini coefficient directly applied to the number of crimes does not change significantly when the distribution changes, which is precisely its main weakness.

The Rare Event Concentration Coefficient *RECC* is easy to compute and provides a summary statistic which is comparable and also, measurements which help us detect structural changes in the dispersion of rare and highly-concentrated events, such as crime, road accidents or human mobility.

### Extensions

The *RECC* is designed for rare events, so, in general we observe many zeros which do not ensure that the rate of the individuals is zero, so if a person, for example, suffered zero crimes last year it does not mean that their rate is equal to zero, and so we assume the simplest possible model, which is a mixture model based on a Poisson distribution.

If events are not as rare, then it is possible to estimate the individual rates using a different technique than the mixture model, which considers information at its full and that tries to mimic the underlying pattern. For example, we can consider the rates at which underground stations serve their users, which is best modelled taking into account the hour and the day of the week, or even the number of mobile users within the nearest routeing tower vicinity, which might be modelled taking into account the time and space. More sophisticated models for a counting process can also be considered, for example, gang shootings may incite retaliation from rival gangs, and an earthquake increases the chances of a second earthquake, causing in both cases a self-exciting process^20^. In that case, since it is not a rare event, estimating the individual rates, either as a function of time, space and/or past events, gives a much better approximation to reality. Thus, the Event Concentration Coefficient (*ECC*) can be constructed simply by computing the Gini coefficient of the individual rates, even in the case in which they were estimated using a different model. The resulting metric provides, as in the case of the *RECC* a number between zero and one which reflects the level of concentration of such events.

## Additional Information

**How to cite this article**: Prieto Curiel, R. and Bishop, S. A measure of the concentration of rare events. *Sci. Rep.* **6**, 32369; doi: 10.1038/srep32369 (2016).

## Figures and Tables

**Table 1:** Observed number of burglaries in Netherlands, 1993.

Number of burglaries	0	1	2	3	4	5	6	7	8	9	10	11+	Total
Number of houses	36,632	2,548	448	134	51	19	8	2	1	2	2	2	39,849
Frequency (%)	91.9	6.4	1.1	0.3	0.1	0	0	0	0	0	0	0	100

**Table 2:** Results of the mixture model applied to the burglaries suffered in the Netherlands.

Group	1	2	3	4	5
Crime rate 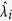	0	0.11	0.30	1.79	7.93
Relative size 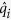 (%)	58.2	24.3	15.8	1.7	0
Number of houses	23,188	9,679	6,282	688	12

*RECC* = 0.7643.

**Table 3:** Volcanic eruptions in the world between 1966 and 2015.

Eruptions	0	1 to 8	9 to 16	17 to 24	25+
Volcanoes	1,217	249	39	20	7
(%)	79.4	16.3	2.5	1.3	0.5
